# Soma Design for Digital Mental Health and Well-Being Interventions: Scoping Review

**DOI:** 10.2196/79400

**Published:** 2026-05-11

**Authors:** Sachiyo Ito-Jaeger, Aysegul Humeyra Kafadar, Steve Benford, Elvira Perez Vallejos

**Affiliations:** 1 Mental Health & Clinical Neuroscience, School of Medicine, University of Nottingham, Innovation Park, Triumph Road, Nottingham, NG7 2TU, United Kingdom, 44 01158231294; 2 National Institute for Health and Care Research (NIHR) Nottingham Biomedical Research Centre, Nottingham, United Kingdom; 3 The Institute for Lifecourse Development, University of Greenwich, London, United Kingdom; 4 School of Computer Science, University of Nottingham, Nottingham, United Kingdom

**Keywords:** scoping review, soma design, digital mental health, well-being, human-computer interaction, embodiment

## Abstract

**Background:**

Digital interventions for mental health and well-being are increasingly moving beyond screen-based applications toward more embodied approaches, necessitating design methodologies that emphasize bodily experiences. Soma design offers a distinctive interaction design approach that integrates bodily awareness with aesthetic appreciation, viewing the mind and body as an inseparable whole.

**Objective:**

This scoping review aims to map and analyze the emerging applications of soma design within digital mental health and well-being (DMHW) interventions, offering a comprehensive overview of this holistic design methodology for researchers and practitioners.

**Methods:**

This review was conducted in accordance with the PRISMA-ScR (Preferred Reporting Items for Systematic Reviews and Meta-Analyses Extension for Scoping Reviews) guidelines. Studies were included if they used soma design to develop DMHW intervention.

**Results:**

Nine papers were eligible for inclusion. The interventions varied in their stage of development: 5 were conceptual design concepts created by adolescents, while 6 were prototypes or experiential artifacts developed by researchers and/or participants. All interventions incorporated soma awareness exercises, with Feldenkrais lessons being the most commonly used. Toolkits, such as Soma Bits and the Menarche toolkit, supported the design of 2 interventions. Soma design methods benefited both designers and users: designers used embodied practices to inform interaction design, while users reported increased bodily awareness, full-body engagement, emotional comfort, and relaxation.

**Conclusions:**

Soma design represents a valuable approach for developing embodied, user-sensitive DMHW interventions. It offers a participatory, holistic co-design methodology that can meaningfully engage end users. However, many interventions identified in this review remain in early stages of development and lack systematic evaluation. Advancing the field will require interdisciplinary collaboration among mental health professionals, human-computer interaction researchers, clinicians, industry partners, and individuals with lived experience. These partnerships are essential for co-designing, testing, and implementing interventions that are both effective and scalable, ultimately extending the reach and impact of soma design in digital mental health contexts.

## Introduction

### Background

#### Overview

The use of digital interventions to support mental health and well-being (MHW) is rapidly expanding [1]. These interventions harness technology to deliver accessible, scalable, and often cost-effective solutions for a wide range of psychological conditions. To date, the most studied digital mental health tools are screen-based, typically delivered through websites and mobile apps [2-4].

However, there is growing interest in more embodied digital interventions, those that engage the body through movement, sensation, and physical interaction. Technologies, such as virtual reality (VR) therapy, socially assistive robots, and wearable devices, are increasingly being explored for their potential to create more immersive and physically engaging therapeutic experiences [5-7]. As the field moves toward more embodied approaches, design methodologies that center bodily experience become increasingly relevant. One such methodology that has gained traction in human-computer interaction (HCI) is soma design, which offers a holistic design stance on embodied interaction that could inform the development of future digital mental health and well-being (DMHW) interventions.

#### Embodiment in MHW

Embodiment refers to the concept that our mental and emotional experiences are deeply intertwined with our bodily sensations and movements [8]. Embodiment interventions are based on the idea that changes in bodily movements or postures can generate or enhance emotional experiences and activate related cognitions [9]. Neuroscientific evidence supports this bidirectional relationship, showing that bodily states, such as facial expressions, hand contractions, and posture, can influence both emotional experience and cognitive functioning [10].

A recent literature review highlights the therapeutic benefits of various embodied approaches in treating mental health symptoms [8]. For example, Dance Movement Therapy reduced depression while it improved well-being and overall health [11-13]. Similarly, relaxation training, such as mindful deep breathing, has demonstrated efficacy in alleviating symptoms of anxiety disorders [14].

Digital mental health interventions are also increasingly incorporating embodied technologies. One prominent example is VR exposure–based treatment, which immerses patients in controlled, interactive environments that facilitate gradual exposure to feared stimuli. This method has demonstrated efficacy in treating conditions such as post-traumatic stress disorder and eating disorders, offering patients the ability to tailor exposure scenarios and modulate their intensity [15-18].

In addition, socially assistive robots have shown potential in supporting MHW. A recent systematic review highlights their promise, though it also underscores limitations in the current body of research, including a narrow scope, limited generalizability, and methodological weaknesses, pointing to the need for more rigorous studies [6].

Wearable technologies are also emerging as effective tools in clinical contexts. For instance, a wrist-worn device that delivers rhythmic electrical stimulation to the median nerve has been shown to significantly reduce the frequency and intensity of tics in individuals with Tourette syndrome, illustrating the therapeutic potential of neuromodulation through wearable interfaces [7].

Overall, the evidence suggests that embodiment practices, both analog and digital, offer valuable contributions to MHW interventions, warranting further exploration and integration into DMHW frameworks.

#### Soma Design

Soma design and embodiment are closely intertwined concepts. Soma design is a distinctive approach within HCI that foregrounds the role of the body in interaction design, integrating bodily experience with aesthetic and sensory engagement. Rooted in the philosophical framework of *somaesthetics*, developed by Richard Shusterman, soma design draws on the concept of the *soma*, the living, sentient body as experienced from within, and *aesthetics*, the cultivation of sensory awareness and appreciation [19]. This framework emphasizes the integration of body, movement, thought, and emotion, placing embodied subjectivity at the center of design [20,21].

Building upon Dewey’s notion that aesthetic experience arises through active engagement rather than being an inherent property of objects [22], Shusterman’s *somaesthetics* encourages a deepened awareness of sensory experience. Soma design translates these philosophical insights into a practical design methodology that challenges traditional mind-body dualisms. It embraces a holistic view of human experience, recognizing that cognition, emotion, and perception are inseparably linked to bodily states and movements [23,24].

This approach invites designers to create technologies that enrich users’ lived, embodied experiences. It does so by incorporating the designer’s own bodily awareness and the physical-digital materials they engage with as integral components of the design process [24-26]. This involves paying attention to how users feel, move, and perceive their interactions with technology and creating designs that enhance these experiences in a harmonious and integrated manner. This integration ensures that the resulting interactive systems/artifacts can effectively influence and enhance the end users’ and the designers’ sensual appreciation and movement experiences [26].

Central to this methodology is the cultivation of somatic awareness through practices, such as body scans [27], Feldenkrais exercises [28], Alexander technique [29], yoga, and other movement-based activities [30-32] (Table 1). These practices help designers attune to their own bodily sensations and develop a first-person understanding of embodied experience, which then informs the creation of interactive systems for others [23,25]. This shift from verbal or cognitive ideation to movement-based exploration enables designers to “do” rather than “talk,” anchoring their process in the subtleties of sensation and presence [21,33].

**Table 1.** Definitions of soma awareness exercises.

Soma awareness exercises	Definitions
Alexander technique	A mindfulness practice and somatic modality that helps people become consciously aware of their habitual movement patterns and posture, with the goal of releasing inefficient or harmful physical habits that create tension and pain
Body scans	A type of mindfulness-based stress reduction exercise in which practitioners systematically direct focused attention to bodily sensations throughout different parts of the body in sequence
Feldenkrais exercises	A somatic movement practice developed by Moshe Feldenkrais that focuses on increasing awareness of habitual movement patterns, through slow, mindful movements guided by a practitioner, aiming to help people discover more efficient ways of moving by reducing unnecessary effort while achieving optimal results. The method helps individuals rediscover alternative movement patterns they may have forgotten, particularly beneficial for those experiencing pain or movement difficulties
Other bodily practices	Other bodily practices include various introspective physical activities and experiences, such as movement exploration, touch awareness, and horseback riding that designers engage with to inform and ground their design work

Philosopher and dancer Maxine Sheets-Johnstone further enriches this perspective by emphasizing the moving, living body as central to how we inhabit and make sense of social, physical, and temporal spaces [34]. Soma design aligns with this view, encouraging designers to engage with the body not as an object, but as a dynamic, expressive medium of experience.

To deepen bodily awareness and disrupt habitual perception, soma design often employs Soma Bits, technologies embedded in soft, body-worn materials that use heat, vibration, or inflation to evoke unfamiliar sensations and prompt reflection (see Table 2) [35,36]. These tools help both designers and users become attuned to the nuances of the embodied interactions.

**Table 2.** Definitions of toolkits.

Toolkits	Definitions
Soma Bits	Soma Bits is a design toolkit consisting of foam shapes covered in elastic cotton textile that can be placed on different body parts, combined with various actuators (heat pads, vibration motors, and pneumatic shape-changing materials) that can be inserted into shapes pockets. The toolkit serves as a living, growing library intended to help designers explore and ideate with smart materials by providing the immediate, direct experience of potential soma designers through different combinations of shapes and actuators on the body.
Menarche Bits	Menarche Bits is a customizable prototyping kit of soft, silicone, body-worn interfaces that respond to pressure through air-actuated chambers. Designed for young adolescents experiencing menarche, it includes both preprogrammed components with haptic feedback and modular pieces that can be interconnected, allowing users to create personalized technologies that help them better understand and trust their menstruating bodies.

Soma design has gained traction in HCI over the past decade. However, its application in DMHW remains underexplored. It is also unclear whether existing Soma-based interventions primarily support general well-being or target specific mental health conditions. Digital mental health is defined as “the application of digital technologies in mental health care which can be used for many purposes, including MHW promotion and prevention, well-being maintenance/self-care, early intervention, or for treating specific mental illness…” [37] While well-being can encompass physical, mental, social, and environmental dimensions [38], in this review, we focus specifically on mental well-being.

### Aims and Research Questions

This scoping review aimed to explore soma design applications in DMHW interventions.

To guide this review, we formulated the following research questions:

- Main question: What DMHW interventions have been designed using soma design methods?
- Subquestions:

1. What soma awareness exercises and other embodied activities have been employed in these interventions?
2. Who participated in the design process?
3. Do these interventions primarily engage general well-being or target specific mental health conditions?
4. What evaluation approaches were used to assess these interventions?
5. What outcomes were reported?

## Methods

### Study Design

The scoping review was conducted to map the existing literature in this research area [39,40], following the 5-stage framework: (1) identifying the research question, (2) identifying relevant studies, (3) selecting studies, (4) charting the data, and (5) collating, summarizing, and reporting the results [39] and the principles of the PRISMA-ScR (Preferred Reporting Items for Systematic Reviews and Meta-Analyses Extension for Scoping Reviews) guidelines (Checklist 1) [41].

### Search Strategy and Data Sources

Two of the authors (SI-J and AHK) conducted initial literature searches relevant to the research question using databases, including Google Scholar. These initial searches were exploratory and aimed to identify relevant literature and refine the search strategy. Based on the relevant literature found during the initial searches, the search terms for the scoping review were discussed and selected during meetings among the authors. The search terms were combined using Boolean operators as follows:

((“somaesthetic*” OR “soma design”) AND (“health” OR “wellbeing” OR “wellness” OR “illness” OR “care” OR “therap*” OR “affect*” OR “emotion*” OR “feeling*”) AND (“digital mental health” OR “DMH” OR “digital technolog*” OR “technolog*” OR “digital health” OR “digital” OR “mHealth” OR “telehealth” OR “telemedicine” OR “electronic health” OR “mobile apps” OR “mobile health” OR “online based” OR “mobile*” OR “eHealth*” OR “electronic mental health” OR “e-mental health” OR “online-based*” OR “internet-based*” OR “web-based*” OR “computer-based”))

Three databases, PubMed, IEEE Explore, and ACM Digital Library, were searched on March 21, 2024, and again on January 18, 2026, and all results were imported into Rayyan [42] for screening. These databases were selected to capture the most relevant literature across health and medical research (PubMed), engineering and technology (IEEE Xplore), and HCI and design (ACM Digital Library). To enhance the comprehensiveness of the review, we additionally searched Google Scholar (first 50 records) and conducted backwards citation tracking.

### Study Selection and Data Extraction

Studies were eligible for inclusion in this review if they met the following inclusion criteria:

1. Concept: digital mental health or well-being interventions designed using soma design methods and explicitly described as targeting or supporting mental health or well-being
2. Context: studies published in the English language, in any country

Studies were excluded from this review if they met any of the following exclusion criteria: review papers, an invitation to a workshop, papers that did not specifically state that the interventions targeted or supported mental health or well-being, interventions that did not involve digital technology, or papers that did not include sufficient details about the intervention (eg, soma design methods used, soma awareness exercises employed, or the purpose of the intervention).

## Results

### Selection of Sources of Evidence

**Figure 1.**
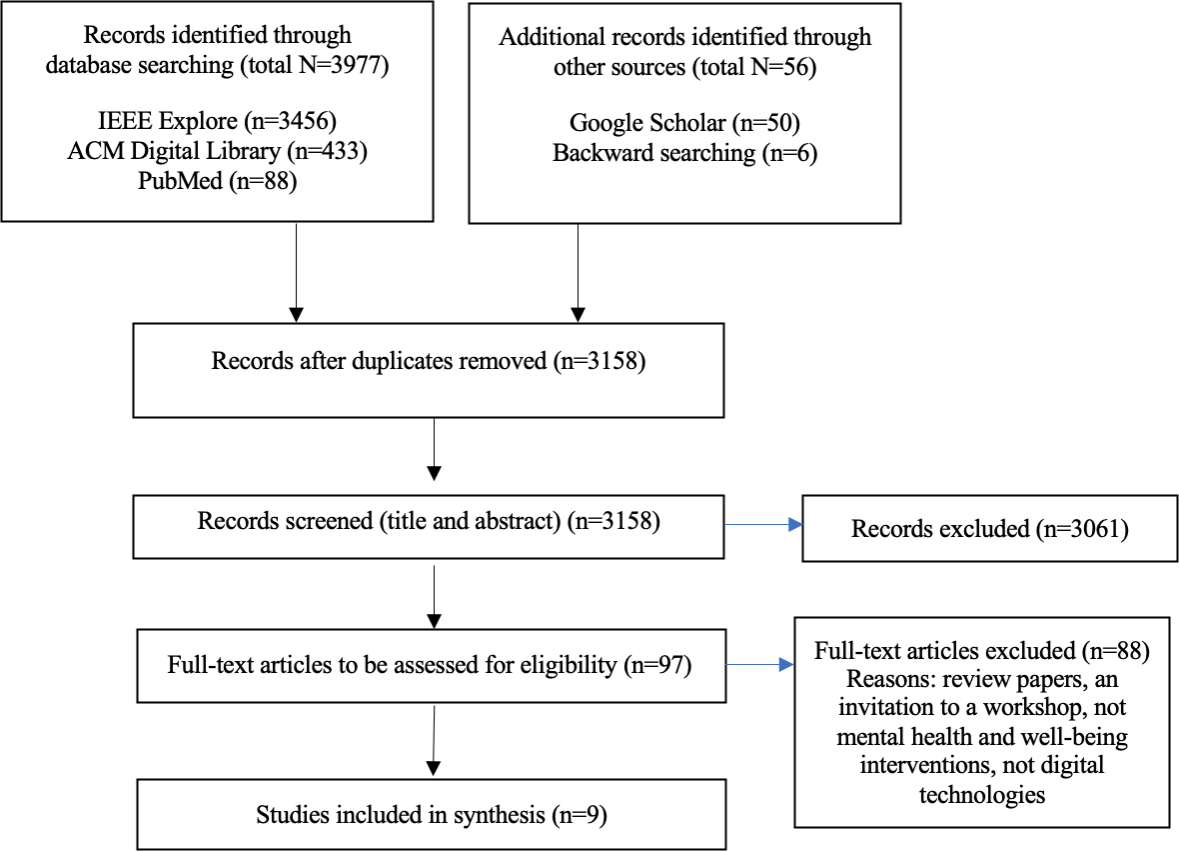
PRISMA-ScR (Preferred Reporting Items for Systematic Reviews and Meta-Analyses Extension for Scoping Reviews) flow diagram of the study selection process.

In total, 3977 articles were identified from the electronic database searches (3456 IEEE Explore, 433 ACM Digital Library, and 88 PubMed), and an additional 56 records were identified through other sources. Following title and abstract screening, 97 articles were selected for full-text review. Two authors (SI-J and AHK) independently reviewed the articles and resolved disagreements through discussion until consensus was reached. Finally, 9 papers met the eligibility criteria and were included in this review (see Figure 1 for the PRISMA-ScR flow diagram).

Several papers described distinct stages or facets of prototype development, while others presented multiple prototypes, design concepts, or experiential artifacts. In total, 11 such items were included in the analysis.

### Data Extraction

Data extraction was conducted independently by 2 of the authors (SI-J and AHK). Extracted information included lead author, year of publication, country of study, digital mental health intervention(s), stage(s) of the product development, MHW framing and timing (ie, whether the importance of MHW was prespecified by designers [predesign] or emerged through collaboration with prospective users and whether mental health conditions or well-being were focused), designers and/or participants, sample size (n), age range, soma awareness exercises and other activities, documentation methods, analytical approaches, and primary outcomes or conclusions (see Tables3  4).

**Table 3.** Characteristics of the sources of evidence and outcomes.

Author, year of publication, and country	Digital mental health intervention(s), stages of the product development, MHW^a^ framing and timing	Designers/ participants and soma awareness exercises and other activities
Ezer et al [43], 2024, Israel	Somaesthetic Meditation Wearable, prototype, predesign (well-being)	Participants, n=5 (varying experience in meditation) A 10-minute meditation session, followed by an interview about their awareness of various body locations. Researchers (experienced meditators) explored various low-fi wearable designs with participants. Participants, n=4 Researchers tested low-fi wearable designs with 4 participants. Participants, n=3 Researchers tested the next design iteration with 3 participants during meditation. Eastern medicine expert Researchers designed the final prototype. Safety and health consultation with Eastern medicine expert. Participants, n=20, age range: 24-59 years 10-minute warm-up. 10-minute meditation: Half of the participants meditated with the wearable and half without. 2-4 minutes reflection sessions. 10-minute meditation session: Half of the participants meditated with the wearable and half without. A postexperience interview.
Höök et al [20], 2016; Stahl et al [35,44], 2016, 2022, Sweden	Soma Mat and Breathing Light, prototypes, predesign (well-being)	Researchers Feldenkrais-lessons once a week for 2 years. Researchers touched, felt, interacted with the materials and created the first prototype. Participants, n­=7 Seven participants tried the prototypes, leading to a range of refinements of the prototype. Participants, n=22, tried the prototype in a 1-hour study: Body map. 30 minutes doing a body scan in the prototypes. Body map. Unstructured interview. Participants in 4 households for 3 months: Use the prototypes 3-5 times a week. Semistructured interview.
Jung et al [45], 2021, Sweden	Deep Touch Pressure (DTP) garment, experiential artifact, predesign (well-being)	Workshops: Researchers, n=5, age range: 23-56 years Four collaborative soma design workshops (3 h each) exploring breathing-based interactions with shape-changing pressure feedback: Feldenkrais practice. Soma Bits. Body maps. Used the drawings to initiate a discussion into how their bodily sensations had changed throughout the session. Design sessions: Researcher, n=1, age=23 years The researcher created a restrictive garment which contains the shape-changing pads, pressing against both the upper and lower back. For 3 weeks, the researcher conducted 1-2 sessions daily for 30-60 minutes each.
La Delfa et al [46], 2020, Australia	Drone Chi, prototype, predesign (well-being)	The principal designer and a Tai Chi master (co-design) The principal designer took regular Tai Chi lessons over 6 months. Consulted the “somatic connoisseurship” of the Tai Chi master who gave the lessons, by involving him in a 2-hour co-design session and engaging with the drones together. Participants: n=32 (13 males, 19 females) Participants tried using the prototype.
Montoya et al [47,48], 2023, Australia	Fluito, prototype, predesign (well-being)	Researchers The main designer interacted with water in different settings over 1 month. The main designer explored different technologies in water settings. Researchers conducted, recorded, and documented three 30-minute sessions over 3 weeks (1 per wk) in their laboratory-installed flotation tank. Slowstorming to combine interactive technology with a flotation tank. Participants: n*=*13 (6 females, 7 males), age ranged from 22 to 65 years (mean 31.8) Float for 10 minutes in the Fluito to get used to the flotation in the tank, Put on the VR^b^ headset in the tank. Semistructured interview 30-45 minutes.
Søndergaad et al [49], 2021, Sweden	Menstrual technologies 5 design concepts by the adolescents *Värme Plagget* (heat garment) *The Calm-inator* *U-formen (The U-shape)* *Korven (The Sausage)* *Transformer* Design concepts, predesigned (well-being: emotional aspects), emerged through collaboration: (anxious and depressive feelings)	Researchers: age ranged from 29 to 38 years from Northern European and South American countries: Recalled and re-enacted their menarche experiences. Increased their bodily awareness of their current bodies in sport activities. Participants: adolescents who do sport and who recently started to menstruate, n=7, age ranged from 16 to 18 years. 4-hour workshops: Drew their menstrual cycles. Wrote down two menstrual memories. Body map. Feldenkrais-inspired lesson to create awareness of breathing and pelvis movements. Body map. Designed a body-worn technology that engages with their unique experience of their menstrual cycle, using Menarche Bits. The participants recorded a short video of themselves presenting their design concepts on their own smartphones.

a MHW: mental health and well-being.

b VR: virtual reality.

**Table 4.** Characteristics of the sources of evidence and outcomes (continued).

Author, year of publication, and documentation methods		Analytical approaches	Primary outcomes/conclusions
Ezer et al [43] (2024)			
Interview		N/A^a^	Low-fi wearable designs Participants prefer the circular shapes and to explore various body locations The need to support easy placements on a variety of body parts No risk Recommended starting with the lower abdomen or lower back
Semistructured interview (questions adapted from the State Mindfulness Scale)		Thematic coding process	The warmth provided emotional comfort
Höök et al [20] (2016); Stahl et al [35,44] (2016, 2022)			
Body map before and after the session Unstructured interview		N/A	Overall, the experience was very positive, and participants expressed that they wanted to have these at home as a relaxation tool
All interviews were transcribed verbatim		Storytelling	Through using the prototypes, the users learned a new experience, which gave them an alternative way of being in the world
Jung et al [45] (2021)			
Workshop: Notes Pictures Videos Soma body sheets/body maps		N/A	These sessions enabled the authors to narrow down the physical schematics that would facilitate meaningful interactions. They included a set of suitable pad shapes/sizes and bodily placements that could be embedded into a wearable interactive system, later finalized by one of the researchers Four experiential esthetic qualities were developed Calm and relaxed feelings
La Delfa et al [46] (2020)			
Embodied sketching (use the body to act out design ideas in situ, capturing them on media like video as they unfold in time)		Applied the method in reflective encounter with the materials	N/A
Video recording Body sheets after the sessions Semistructured interviews		Thematic analysis	Initially the hands were demanding the most attention. By the second session, most participants were moving more fluidly, coordinating the whole body to produce smooth moving hands Feeling relaxed
Montoya et al [47,48] (2023)			
Body maps, written descriptions, and video recording before, after, and where possible during these experiences		N/A	N/A
Semistructured interview		Inductive thematic analysis	Four themes, including “Fluito as a facilitator for relaxation.” All participants reported relaxation due to additional reasons relating to the interactive technology.
Søndergaad et al [49] (2021)			
Researchers: Video recording Notes Photos Participants: Body maps A-lab sheets Phone video recording		Transcribed the participants’ videos and the key moments from the video recording Wrote thick descriptions	The participants designed five technologies that respond to their experiences of menstrual cycles in sport. The design concepts demonstrate interactions with breathing, touch, modularity, and rhythms on intimate placements on the body, including the pelvis bone and chest Throughout the workshop, the participants’ accounts of menstrual cramps and depressive and anxious feelings went from linguistic to embodied.

a N/A: not applicable.

### Stages of the Product Development

The products described across the included papers varied considerably in their stage of development. Some were fully developed prototypes created by researchers and evaluated by participants, while others represented earlier phases of the design process, such as design concepts or experiential artifacts. *Design concepts* refer to bodily informed, experience-driven ideas for potential future products. *Experiential artifacts* are intentionally designed systems that elicit specific experiences based on the properties of a chosen digital material, serving as exploratory tools for design teams rather than goal-oriented, deployable products [50]. Throughout this paper, we use the terms, *prototype, design concept, or experiential artifact*, to accurately reflect the development stage of each product. When discussing collectively, we use the term *interventions* for clarity and readability.

Eleven DMHW interventions created using soma design methods were found. The *Soma Mat* is a body-sized mat, which directs the user’s attention by providing heat feedback to different parts of their body while they follow the instructions of a prerecorded Feldenkrais lesson [20,35,44]. The authors recommend that the *Soma Mat* should preferably be used with another intervention, the *Breathing Light* [20,35,44]. The *Breathing Light* is a lamp that measures the user’s breathing, creating an ambient light that dims in cadence with their breathing. The user is recommended to lie on the *Soma Mat* while placing the *Breathing Light* above their face to provide them a private space.

The *deep touch pressure (DTP) garment* is an experiential artifact created to help increase breathing awareness and appreciation through interaction with breathing-based deep pressure [45]. *Fluito* has a combination of technology (a flotation tank, a VR headset, a heart rate sensor, and a pneumatic system), which creates different experiences for the user, including relaxation [47,48]. *Drone Chi* is inspired by the martial art of Tai Chi, providing a close-range human-drone experience by the user either following or leading the drone [46]. The *Somaesthetic meditation wearable* is a novel targeted warmth wearable intended to be used during meditation [43].

Adolescent athletes developed 5 innovative menstrual technology concepts to address menstrual cramps and premenstrual depressive and anxious feelings [49]. Using Menarche Bits [49], participants designed technologies that incorporate touch, breathing rhythms, and modularity in intimate body placements (see Table 2). *Värme Plagget* (heat garment) is a wearable garment that provides heat to alleviate menstrual pain in the lower abdomen. *The Calm-inator* is a blouse equipped with air pumps that massage the chest area, helping to increase breathing awareness. *U-formen (The U-shape)* consists of 2 heat cushions that warm the lower abdomen and pumps that create pulsating sensations on the hip bones. *Korven (The Sausage)* is a portable heat cushion to relieve menstrual pain and bloating before and during menstruation. *Transformer* consists of heat and pumping machines, which can be placed in the middle of the stomach to ease menstrual pain.

### MHW Framing and Timing

Across most interventions, MHW considerations were prespecified by the designers, typically framed around goals, such as relaxation or calmness, rather than clinical treatment. In one study, the relevance to MHW emerged during collaboration with prospective users, although emotional dimensions had already been implied by the authors [49]. Overall, all included interventions focused on general well-being rather than addressing specific mental health conditions.

### Designers

Five of the interventions were designed primarily by researchers. At the time of the papers’ publication, 1 intervention (*DTP garment*) had been developed solely by the research team [45], whereas 4 interventions (*The Soma Mat*, *The Breathing Light, Drone Chi*, and *Fluito*) were first designed by researchers and subsequently tried by participants, who provided experiential feedback [20,46,47].

The *Somaesthetic Meditation Wearable* was designed by participants with varying experience in meditation [43]. The 5 menstrual technologies were designed by adolescents who played sports [49]. Specialist expertise was integrated into 2 studies: one study involved a “somatic connoisseur,” a consultant specializing in a particular bodily practice (in this case, a Tai Chi master) who contributed to a co-design session [46], whereas the other consulted an expert in Eastern medicine during the design process [43].

### Soma Awareness Exercises and Toolkits

#### Soma Awareness Exercises

The soma awareness exercises practiced most frequently during the design processes were Feldenkrais lessons. Researchers practiced Feldenkrais lessons before designing 3 of the interventions: *Soma Mat, Breathing Light, and DTP garment* [20,45], while adolescents practiced them before creating the 5 design concepts of the menstrual technologies [49]. The types of Feldenkrais lessons varied depending on the focus of each project. Other soma awareness exercises tried by the designers included Tai Chi, meditation, interacting with water, and engaging in sports [43,46,47,49].

#### Toolkits

The *Soma Bits Toolkit* and the *Menarche Bits* were specifically designed and created for soma design practice [26,51]. The *Soma Bits* were used to design the *DTP garment* [45], whereas the *Menarche Bits* were used by adolescents to design the 5 menstrual technologies [49] (see Table 2).

### Documentation Methods

In addition to taking notes during workshops and conducting participant interviews, researchers employed a range of strategies to document the soma experiences encountered during the design process. To capture how designers or participants felt in their bodies, each person filled out the body maps [52] before and after bodily activities in 8 studies (Table 5). These body maps were filled out by researchers in the development of 2 interventions (*DTP garment* and *Fluito*) [45,47], by adolescents during the co-design of 5 menstrual technologies [49], and by participants to report their experiences with 3 of the interventions: *Soma Mat, Breathing Light,* and *Drone Chi* [20,35,44,46].

**Table 5.** Definitions of body maps and experiential qualities.

Documentation	Definitions
Body maps	Body maps are tools for body awareness consisting of preprinted body silhouettes on paper that can be annotated and drawn upon. They serve as a method to document and reflect on one’s lived bodily experience, allowing individuals to externalize and track changes in body awareness, particularly before and after somatic practices, such as Feldenkrais. By sharing these visual representations, body maps help develop a more refined vocabulary for describing bodily experiences while fostering trust and comfort among participants.
Experiential qualities (XQs)	XQs are characteristics embedded in a design and felt during user interaction, serving as guiding principles throughout the design process while allowing creative freedom in translating somatic experiences into multiple design ideas and materials.

Researchers also frequently used video recordings to document key moments during the design process and interviews. For 3 interventions, such as *DTP garment, Drone Chi,* and *Fluito*, researchers recorded soma design workshops to reflect on their own experiences [45-47]. Additional recordings captured participants using the *Drone Chi* intervention [46] and adolescents engaging in the design of menstrual technologies [49]. In one study, participants were asked to record short videos on their phones to present their design concepts (menstrual technologies) [49].

### Analytical Approaches

In all studies, designers engaged in first-person, subjective reflection to foreground experiential insights, facilitate iterative exploration, and foster reflective practice throughout the design process. To analyze the data collected from participants, a range of qualitative methods was employed. Thematic analysis was conducted to analyze semistructured interviews with participants for 3 of the interventions, such as *Somaesthetic Meditation Wearable*, *Drone Chi*, and *Fluito*, to identify recurring patterns and themes [43,46,47]. For the *Soma Mat* and *Breathing Light* interventions, a storytelling approach was adopted to capture and interpret individual participant experiences [35]. In many of the studies, the use of video data, which captured the moments from the design process or participant interviews, was not explicitly detailed. However, in the case of *Drone Chi*, video content was integrated into the interviews and analyzed thematically [46]. For the menstrual technologies project, researchers produced thick descriptions of key workshop moments to support interpretive analysis [49].

### Outcomes

#### For Designers Developing the Interventions

Designers reported a range of outcomes from applying soma design methods in the development of 7 interventions. In the case of the *DTP garment*, engagement in soma-based activities, such as Feldenkrais practice and body mapping, enabled the designers (ie, researchers) to identify physical schematics that would support meaningful interaction. These practices also helped uncover key experiential qualities [53] (see Table 5) that informed the design of the intervention [23,45].

Similarly, adolescent designers who participated in a soma design workshop to develop menstrual technologies experienced a shift in how they articulated their symptoms. Their descriptions of menstrual cramps, depression, and anxiety evolved from linguistic expressions to embodied understandings. Through this process, they began to perceive their bodies and minds not as separate entities, but as interconnected aspects of their lived experience. Their resulting design proposals aimed to alleviate menstrual discomfort by incorporating pain relief and breathing techniques to address both physical and emotional symptoms [49].

#### For Participants Experiencing the Interventions

Five of the 11 interventions developed using soma design were evaluated by participants who had not been involved in their creation. Overall, these participants reported positive and meaningful experiences when engaging with the interventions. For instance, users of *Fluito* described an increased awareness of their breathing, which contributed to both mental and physical relaxation [47]. Similarly, participants interacting with *Drone Chi* demonstrated a gradual shift in their engagement. Initially focused on hand movements, they began to move more fluidly over time, incorporating their entire bodies to produce smooth, coordinated gestures. Analysis of body maps, video recordings, and interviews revealed that *Drone Chi* supported a range of mental states, including meditative experiences characterized by present-moment focus and relaxation [46]. Participants using *Somaesthetic Meditation Wearable* during meditation reported that the warmth provided emotional comfort [43].

Participants who used the *Soma Mat* and *Breathing Light* for 30-minute sessions also reported positive experiences and expressed a desire to use these tools at home for relaxation. Notably, after 3 months of home use, participants reported being able to self-regulate and calm themselves during stressful moments, even without the physical presence of the prototypes, suggesting a lasting impact on their embodied coping strategies [35].

## Discussion

### Current Evidence for Soma Design in DMHW

This scoping review mapped the current landscape of DMHW interventions developed using soma design methods. All included studies reported positive outcomes associated with soma design. Among the 5 interventions tested by participants, users reported increased bodily awareness, full-body engagement, enhanced emotional comfort, and relaxation. Notably, 2 of the interventions demonstrated lasting effects, with participants applying the skills they had developed, such as self-regulation and relaxation, beyond the duration of the intervention sessions. Designers also reported significant benefits from using soma design. These included the identification of experiential qualities that informed the design process and a shift in how adolescent designers perceived their bodies, not as separate from the mind, but as integrated aspects of their whole being. These findings suggest that soma design can offer meaningful value for both designers and users.

However, the balance of evidence is inconclusive due to the heterogeneity in the stage of design development (ranging from conceptual designs to functional prototypes), the identity of the designers (eg, researchers vs adolescents), the extent of end-user involvement, and the analytical approaches employed. In the following section, we explore both the opportunities and challenges in advancing soma design within the DMHW field.

### Opportunities and Challenges in Advancing Soma Design for DMHW

This scoping review highlighted the value of mind-body-focused approaches within DMHW, drawing attention to the often-overlooked role of bodily awareness in design processes. While much of DMHW has traditionally centered on cognitive and screen-based interventions, soma design underscores the importance of attending to the body as a site of both experience and intervention [54]. It adopts a first-person, subjective stance in which the designer’s own bodily experience becomes a source of insight and knowledge production [33]. Grounded in the principles of Research through Design, soma design values reflective practice, iterative making, and somatically grounded engagement with materials and technologies [21]. These epistemological foundations not only distinguish the soma design methodologically but also enable the development of more nuanced, embodied, and user-sensitive DMHW interventions.

In doing so, soma design may offer a bridge between 2 emerging trends in health care: digital mental health and digital physical health. While these domains have often evolved in parallel, one focusing on psychological well-being through digital therapeutics, the other on physiological monitoring and movement-based interventions, soma design invites a more integrated perspective. It encourages the design of technologies that do not treat the mind and body as separate domains but instead recognize their continuous interplay [24]. This integration is particularly relevant in conditions where mental and physical symptoms co-occur [55]. By fostering embodied awareness and sensory attunement, soma design opens up new possibilities for interventions that are not only technologically innovative but also deeply attuned to the lived, felt experience of users. In this way, it contributes to a more holistic vision of digital health, one that supports the whole person, rather than isolating mental or physical aspects of well-being.

This integrative approach aligns with a broader movement within HCI and DMHW toward more body-centric technologies and interaction paradigms. Similar to HCI, the DMHW field is increasingly moving beyond traditional screen-based or VR-focused interventions and embracing emerging modalities, such as wearables, soft robotics, and other forms of embodied interaction that integrate seamlessly with the body [54]. As such, soma design contributes to ongoing conversations about the future of DMHW, suggesting that embodied, somatically informed design practices may play a critical role in shaping more holistic and responsive mental health technologies.

This holistic vision is operationalized through a range of embodied design techniques that lie at the heart of soma design practice. These include soma awareness exercises, body mapping exercises, and the use of Soma Bits, technologies that deliver unfamiliar sensations through heat, vibration, or inflation to disrupt habitual perception and foster deeper bodily engagement [54]. These methods serve as generative design strategies, enabling participants and designers to access and articulate subtle aspects of embodied experience. In doing so, they help ground the design process in lived bodily sensation, reinforcing soma design’s commitment to creating technologies that are attuned to the full spectrum of human experience.

### Cocreation and Stakeholder Engagement

While soma design offers a compelling framework for creating more holistic and embodied DMHW interventions, realizing its full potential depends on meaningful engagement with the people that these technologies are intended to support. However, the studies reviewed revealed varying degrees of user participation. Only 2 studies involved end users from the onset, resulting in the development of 1 prototype and 5 distinct design concepts. In contrast, others engaged participants only during later testing phases.

In mental health research, cocreation is widely recognized as a cornerstone of effective intervention design. It involves engaging end users as active stakeholders throughout the design, development, implementation, and evaluation of digital interventions [56]. This approach aligns with the principle of responsible research and innovation, which emphasizes the importance of creating technologies that are ethically sound, socially desirable, and aligned with users’ values and needs [56].

Participatory soma design holds significant potential as an embodied, holistic coproduction approach. While cocreation has gained traction in mental health research, it has predominantly focused on cognitive-based interventions, often through patient and public involvement workshops or focus groups. Participatory soma design methods present new opportunities for mental health research by offering a more embodied and experiential approach.

This is particularly important when designing for populations that differ from the researchers themselves. For example, Søndergaard et al [49] demonstrated the value of involving adolescents in the design of interventions targeted at their age group. Similarly, including individuals with lived experience of neurodiversity or mental health conditions is essential to ensure that interventions are both relevant and effective.

However, the time- and resource-intensive nature of soma design can make sustained user involvement challenging. Ideally, end users should be engaged from the early stages of design. Where this is not feasible, researchers should still involve users in later stages of refinement to ensure that interventions align with their embodied needs and preferences. Adapted or phased models of participation may help balance feasibility with meaningful engagement.

Addressing these challenges requires not only thoughtful engagement strategies but also broader collaboration across disciplines and stakeholders to support the development and implementation of effective soma design interventions.

### Collaborations to Create Effective Mental Health Interventions for the Users

While soma design offers a compelling framework for embodied DMHW interventions, its broader impact relies on the ability to translate rich, experiential practices into solutions that are scalable and accessible. Despite its potential to support MHW in diverse contexts, the field currently lacks systematic evaluation frameworks to assess the effectiveness of these interventions. Much of the existing research remains exploratory, limiting its integration into DMHW practices that require rigorous validation and replicability.

Additionally, this review found a notable distribution between mental health conditions and well-being: no included intervention explicitly targeted a specific mental health condition (eg, anxiety and depression). Instead, current applications predominantly focus on well-being outcomes (eg, relaxation and calm). Addressing this gap is essential for understanding the therapeutic potential of soma design and guiding its future applications in both clinical and everyday settings. The interventions currently available may inform the development of future condition–targeted digital mental health interventions.

Furthermore, the interventions reviewed in this study varied widely in their stage of development, ranging from early-stage design concepts to advanced prototypes undergoing iterative testing. In many cases, the primary focus was on exploring and refining the methodological aspects of soma design rather than producing interventions intended for broader deployment. While this has contributed valuable insights into embodied design practices, it has also limited the immediate applicability of soma interventions in real-world mental health contexts. Nevertheless, examples, such as *Soma Mat* and *Breathing Light* [20], demonstrated the potential of soma design to deliver meaningful, positive experiences to a broader audience. These examples illustrate how embodied interaction can support well-being through sensory engagement and bodily awareness.

To fully realize this potential, interdisciplinary collaboration is essential. Bringing together mental health professionals, HCI researchers, industrial partners, and individuals with lived experience can foster the co-design, evaluation, and implementation of interventions that are both effective and scalable. Such partnerships ensure that interventions are grounded in robust design methodologies while remaining responsive to diverse user needs and real-world constraints. In particular, deepening collaboration with clinical partners and mental health researchers will be crucial for moving soma design beyond exploratory prototypes toward clinically relevant, deployable DMHW solutions.

### Strengths and Limitations of This Review

This paper presents the first scoping review to systematically map existing research on DMHW interventions developed using soma design methods. To our knowledge, it is also the first to introduce soma design to the digital mental health research community in a structured and comprehensive manner. A rigorous literature search was conducted across 3 major databases, supplemented by Google Scholar screening and backward citation tracking to ensure comprehensive coverage. Study selection and data extraction were performed in duplicate to enhance reliability. Through this process, this review offers a valuable synthesis of current knowledge and highlights the potential of soma design to contribute novel, embodied approaches to MHW research.

Despite these strengths, several limitations should be acknowledged. First, the review included only studies published in English, which may limit the cultural and geographical diversity of the findings. This narrow scope may overlook relevant work published in other languages. Second, the included studies varied widely in terms of methodological detail and reporting quality, which may affect the consistency and comparability of findings. Finally, while this review maps the current landscape of soma design in digital mental health, it does not assess the clinical effectiveness of soma design interventions, as no included studies have conducted formal evaluations.

Future research should address these limitations by involving more diverse populations of designers and participants, including individuals from underrepresented cultural and linguistic backgrounds as well as those with lived experience, such as people with mental health conditions or neurodivergence. Broadening both the methodological and demographic scope of soma design research will be essential for realizing its full potential and ensuring its relevance and effectiveness in digital mental health contexts.

### Conclusions

This scoping review provides the first comprehensive synthesis of DMHW interventions developed using soma design methods. The findings suggest that soma design offers a distinctive, embodied approach that can enrich both the design process and user experience by fostering bodily awareness, relaxation, and meaningful engagement.

At the same time, the review identifies important gaps in current literature, particularly around evaluation practices, user involvement, and the diversity of design contexts. Addressing these gaps will be essential for advancing soma design from an exploratory methodology to a widely applicable framework for DMHW innovation.

Looking forward, soma design presents a valuable opportunity to reimagine how DMHW interventions are conceptualized and cocreated. By fostering deeper collaboration among mental health researchers, HCI practitioners, clinicians, industry partners, and individuals with lived experience, future work can support the development of more inclusive, effective, and embodied mental health technologies. In doing so, soma design may help shape a more holistic and human-centered future for digital mental health.

## Supplementary material

10.2196/79400 Checklist 1 PRISMA-ScR checklist.

## Abbreviations

DMHW: digital mental health and well-being
DTP: deep touch pressure
HCI: human-computer interaction
MHW: mental health and well-being
PRISMA-ScR: Preferred Reporting Items for Systematic Reviews and Meta-Analyses Extension for Scoping Reviews
VR: virtual reality
